# Behavioral and Antennal Responses of *Tribolium confusum* to *Varronia globosa* Essential Oil and Its Main Constituents: Perspective for Their Use as Repellent

**DOI:** 10.3390/molecules26154393

**Published:** 2021-07-21

**Authors:** Cesar Auguste Badji, Jean Dorland, Lynda Kheloul, Dimitri Bréard, Pascal Richomme, Abdellah Kellouche, Claudio Roberto Azevedo de Souza, Antônio Lourenço Bezerra, Sylvia Anton

**Affiliations:** 1 IGEPP, INRAE, Institut Agro, University Rennes, CEDEX, 49045 Angers, France; cesar.badji@ufape.edu.br (C.A.B.); jean.dorland@agrocampus-ouest.fr (J.D.); 2 Universidade Federal do Agreste de Pernambuco, Avenida Bom Pastor, s/n.º, Boa Vista, Garanhuns CEP 55292-270, PE, Brazil; clauzzius@gmail.com (C.R.A.d.S.); lourencoagronomia@gmail.com (A.L.B.); 3 Laboratoire de Production, Sauvegarde des Espèces Menacées et des Récoltes, Influence des Variations Climatiques, Faculté des Sciences Biologiques et des Sciences Agronomiques, Université Mouloud Mammeri, Tizi-Ouzou 15000, Algeria; kheloul-lynda@hotmail.fr (L.K.); kader_kellouche@yahoo.fr (A.K.); 4 Univ. Angers, SONAS, SFR QUASAV, F-49000 Angers, France; dimitri.breard@univ-angers.fr (D.B.); pascal.richomme@univ-angers.fr (P.R.)

**Keywords:** stored insect pest, caatinga plant, olfactometry, electroantennogram, alternative pest control, essential oil, repellent effect, aggregation pheromone

## Abstract

Essential oils of aromatic plants represent an alternative to classical pest control with synthetic chemicals. They are especially promising for the alternative control of stored product pest insects. Here, we tested behavioral and electrophysiological responses of the stored product pest *Tribolium confusum*, to the essential oil of a Brazilian indigenous plant, *Varronia globosa,* collected in the Caatinga ecosystem. We analyzed the essential oil by GC-MS, tested the effects of the entire oil and its major components on the behavior of individual beetles in a four-way olfactometer, and investigated responses to these stimuli in electroantennogram recordings (EAG). We could identify 25 constituents in the essential oil of *V. globosa*, with anethole, caryophyllene and spathulenole as main components. The oil and its main component anethole had repellent effects already at low doses, whereas caryophyllene had only a repellent effect at a high dose. In addition, the essential oil abolished the attractive effect of the *T. confusum* aggregation pheromone. EAG recordings revealed dose-dependent responses to the individual components and increasing responses to the blend and even more to the entire oil. Our study reveals the potential of anethole and the essential oil of *V. globosa* in the management of stored product pests.

## 1. Introduction

Pest control in agriculture is one of the most important concerns for producers. Cereal storages provide a special environment that favors insect attack, e.g., by beetles such as *Tribolium confusum*. These beetles are cosmopolitan pests of stored foodstuffs and rank among the most important insect pests of wheat flour and other cereal products. The global damage caused by this insect is about 5% to 30% of the total worldwide production [1]. The most widespread control method is the use of synthetic insecticides, which causes many problems such as contamination of food, environmental pollution, and the emergence of insect resistance [2]. Therefore, the development of alternative, sustainable strategies to fight stored product pests is of utmost importance to reduce the impacts on human and animal health, as well as on the environment. One approach for alternative pest management is to manipulate insect behavior, using olfactory cues.

Many insect species rely on olfactory signals for survival and reproduction. Volatiles emitted by con-specifics guide insects to find a mating partner or to aggregate in order to optimally exploit a resource [3,4,5]. Plant volatiles are used by insects to find food sources and oviposition sites. Responses to volatiles are, however, dependent on a variety of factors, such as previous exposure or the physiological state of the insect [6]. In addition, behavioral responses depend on the olfactory environment and interactions between odors from different sources, which can modify them [7]. Especially interactions between different compounds are important to be considered in the development of semiochemical-based pest management strategies, since certain plant volatiles can reduce the attractiveness of pheromones and, inversely, pheromones can reduce repulsiveness of plant volatiles or synergize with attractive plant volatiles [8,9].

In Brazil, the country with the highest plant biodiversity in the world, and where many species are still unknown to science [10], volatiles emitted by endemic plants may affect insect behavior. The essential oils of plants are a promising alternative to synthetic insecticides because they contain many different volatile compounds, and some of them in large quantities [11]. The main chemical classes of volatile organic compounds present in plant essential oils are terpenes, phenol-derived aromatic components and aliphatic components, and their bioactivity is frequently a result of synergy among constituents [12]. Recently, synergistic effects of essential oil compounds or mixtures of essential oils have been demonstrated for various types of bioactivity, including antioxidant, antimicrobial, and insecticide effects [13,14,15].

In addition to toxicity, antifeedant, and growth-inhibiting effects, essential oils can have attractive or repellent effects on many insects, including stored-product pests [16]. The essential oil of *Pistacia lentiscus,* for example, has a significant repellant effect on major insect pests of stored food such as *Rhyzopertha dominica*, *Sitophilus zeamais,* and *T. confusum* [17]. Different essential oils from wooden plant species, such as *Croton anisatum* and *Illicium verum* are repellent to *Callosobruchus chinensis* [18] and the essential oil of *Lavendula spica* has a repellent effect on *T. confusum* [9].

*Varronia globosa* (Boraginaceae), a shrub that grows in tropical areas of America, is a medicinal plant that is traditionally used for the treatment of diverse illnesses [19]. Various biological activities have been reported for *V. globosa,* such as antibacterial and antifungal properties [20], as well as larvicidal activity [21].

The objectives of the current study were to identify the components of the essential oil from the *V. globosa* leaves using gas chromatography–mass spectrometry (GC-MS) and to test the antennal and behavioral responses to this oil and two of its major compounds individually and as a blend in adults of the confused flour beetle, *T. confusum,* using Electroantennogram recordings and a four-way olfactometer. We also tested the attraction of *T. confusum* to conspecific aggregation pheromone and behavioral responses to simultaneous exposure with a highly repellent dose of *V. globosa* essential oil, its major compounds and *T. confusum* aggregation pheromone to evaluate potential interactions between attractive and repulsive stimuli. Our results show that the *V. globosa* essential oil and its major compounds anethole and caryophyllene are detected by the *T. confusum* antennae and have a repellent effect. They may thus be considered for the development of potential alternative pest management approaches.

## 2. Results

### 2.1. Chemical Analysis of V. globosa Essential Oil

In the chromatogram of the essential oil, 35 peaks occurred and 21 of them could be identified. Approximately half of the identified compounds are propenyl phenols, the other half sesquiterpenes, originating from different metabolic pathways. The most abundant compound was anethole (41.53%) and the second most abundant compound was caryophyllene (7.72%) all other compounds were present in lower amounts (Table 1). We therefore chose these two identified compounds to be tested individually in behavioral and EAG experiments. The third most abundant compound (spathulenol 7.06%) was not commercially available for tests.

### 2.2. Strong Repulsive Behavioral Effect of V. globosa Essential oil on T. confusum

**Figure 1:**
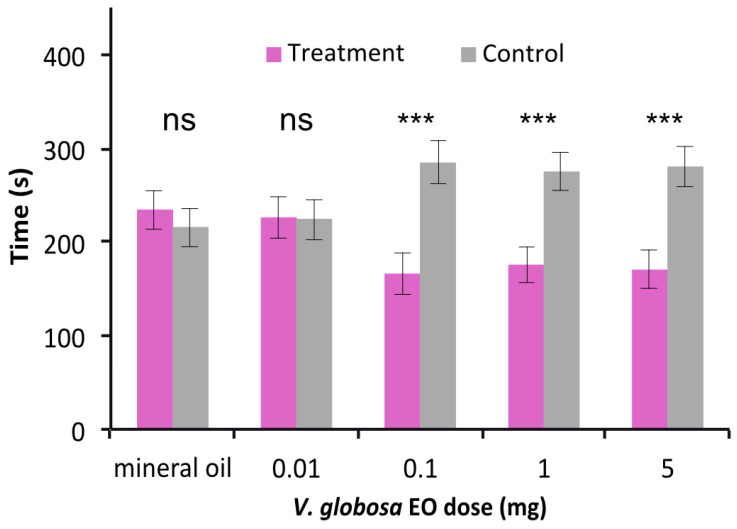
Behavioural effect of *V. globosa* essential oil at different doses in choice experiments against a control (mineral oil) (means, SE). Doses of at least 0.1 mg are repulsive to *T. confusum*. n = 29–32; ns not significant, *** *p ≤* 0.001.

When testing the behavioral choice between all four branches with the solvent mineral oil, no significant preference was observed (*p* = 0.932), indicating that there is no bias for certain branches in our experiments. The *V. globosa* essential oil did not elicit a significant choice at the lowest tested dose of 0.01 mg (*p* = 0.958), but all other tested doses had a highly significant repellent effect on *T. confusum* (*p* < 0.001, *p* = 0.001, *p* = 0.001 for 0.1 mg, 1 mg and 5 mg, respectively) (Figure 1).

### 2.3. Differential Repulsive Behavioral Effects of Main Constituents of V. globosa Essential Oil and Their Mixture

**Figure 2:**
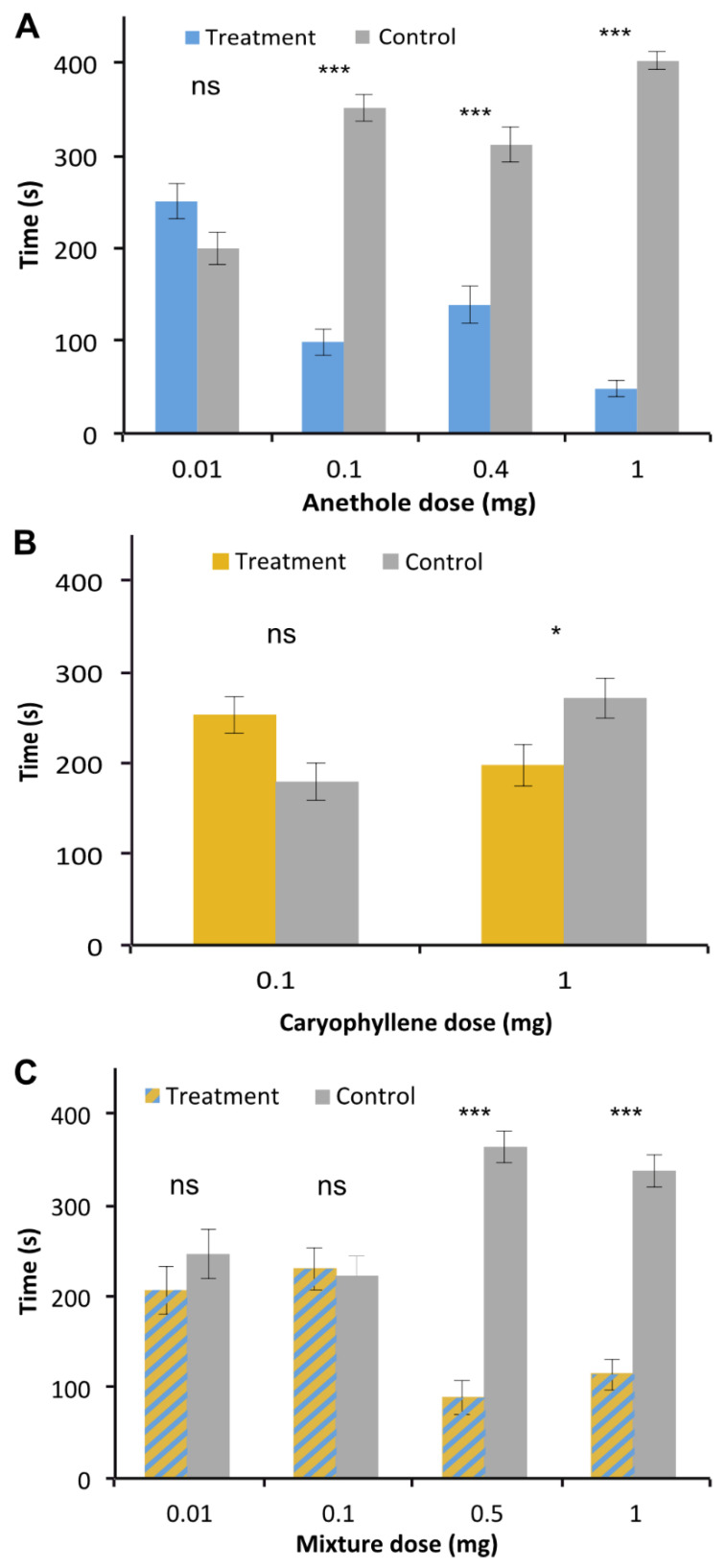
Behavioral effects of major individual compounds of the *V. globosa* essential oil and their mixture. (**A**) Responses to anethole. (**B**) Responses to caryophyllene. (**C**) Responses to the mixture of anethole and caryophyllene. n = 28–31; ns, not significant, * 0.05 > *p* > 0.01, *** *p* ≤ 0.001.

The major compound of the *V. globosa* essential oil, anethole, had no significant effect at 0.01 mg (*p* = 0.07), but had a repulsive effect from an amount of 0.1 mg and higher (*p* ≤ 0.001 for all doses) (Figure 2A). Caryophyllene alone, however, was only significantly repulsive at 1 mg (*p* = 0.011) but not at 0.1 mg (*p* = 0.058) (Figure 2B). The mixture of the two compounds at the ratio of occurrence in the essential oil had no significant effect at the 0.01 and 0.1 mg doses (*p* = 0.289 and 0.815, respectively), but was significantly repellent at 0.5 and 1 mg doses (*p* < 0.0001 for both) (Figure 2C).

### 2.4. Inhibition of Behavioral Aggregation Pheromone Attractiveness by V. globosa Essential Oil

**Figure 3:**
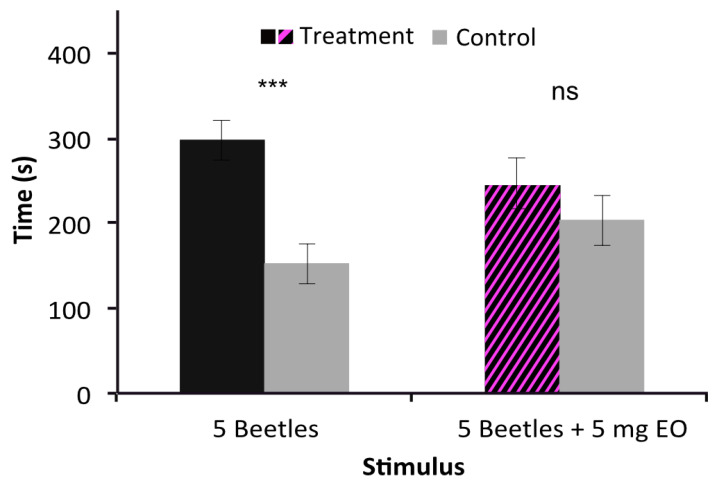
Behavioral responses to the aggregation pheromone alone (5 beetles) and in combination with a repulsive dose of the essential oil (EO). ns, not significant, *** *p* < 0.001.

Five beetles in a stimulation tube were highly attractive to individual *T. confusum*, confirming earlier results [9] (Figure 3, *p* < 0.0001). When combining this attractive stimulus with the highly repulsive dose of 5 mg of the essential oil of *V. globosa*, attractiveness was lost (*p* = 0.308).

### 2.5. Differential Antennal Responses to V. globosa Essential Oil, Individual Constituents and Their Mixture

**Figure 4:**
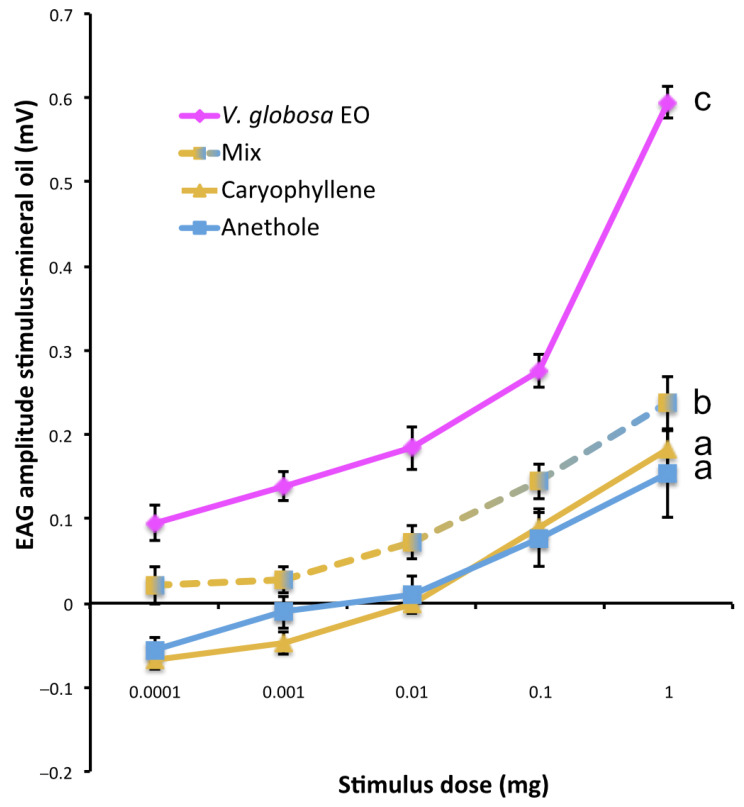
Dose-response curves of EAG responses to the essential oil (EO), individual constituents and their mixture (Mix). Responses to the solvent mineral oil were subtracted (means and SE). Dose-response curves with different letters are significantly different (ANOVA followed by Tukey’s post hoc test *p* < 0.0001). n = 17 for anethole, caryophyllene and mix, n = 11 for *V. globosa* EO.

Both individual compounds were detected by beetle antennae at doses of 100 µg and above on a filter paper. There was no significant difference in antennal sensitivity between the two compounds (*p* = 0.684) (Figure 4). The blend of the two compounds (mix) elicited EAG responses at lower doses (*p* < 0.0001 for both mix vs. anethole and mix vs. caryophyllene) and the complete oil elicited still significantly higher responses (*p* < 0.0001 for mix vs. complete oil) (Figure 4), indicating a physiological role of additional compounds.

## 3. Discussion

Plant bioactive compounds can be used in pest management and reduce the dependence on synthetic pesticides. For essential oils, behavioral responses can be induced by the whole product, by individual compounds or by the combination of part of the compounds. Here, we extracted and analyzed essential oil of the leaves of *V. globosa*. Subsequently, we investigated behavioral responses and antennal detection of *T. confusum* to the essential oil of *V. globosa* and its major compounds.

*V. globosa* essential oil extraction resulted in an extremely low yield. As we only recovered a small amount of oil, the yield was not evaluated. We focused on the composition of the collected oil because, as reported in some Boraginaceae species, the essential oil yield based on the dry weight, is small (less than 1%) in this family [22,23,24,25]. The analysis of the chemical composition of the tested *V. globosa* oil from leaves revealed that anethole and caryophyllene are the major components, followed by spathulenol. Anethole is a compound found abundantly in plants of different families such as fennel and salvia [26,27].

Our experiments on *T. confusum* adults show that they can detect *V. globosa* essential oil and present a dose-dependent behavioral response. Antennal detection, as well as repulsive behavioral responses of *T. confusum*, to the entire oil, to different degrees to its major components, anethole and caryophyllene, and their mixture were discovered. A similar repellent effect on *T. confusum* adults had been observed earlier with *Lavandula spica* essential oil and its main component linalool [9]. In addition to the repellent effect found in the present study, anethole also showed significant toxicity by fumigation against *T. confusum* and *Sitophilus oryzae* adults [28], as well as repellency to two *Tribolium* and two *Sitophilus* species [29]. Additionally, caryophyllene has been reported to have a repellent effect on *T. castaneum* [30].

We also found an inhibition of the attraction by *T. confusum*-produced aggregation pheromones by *V. globosa* essential oil, similarly to *L. spica* essential oil and linalool [9]. A high dose of the essential oil of *V. globosa*, 5 mg on the filter paper, abolished the attraction of the aggregation pheromone emitted by five conspecifics. Essential oils and their major components were also reported to reduce the male attraction to female sex pheromone in *Callosobruchus chinensis* [18]. This is in line with behavioral studies in *Drosophila melanogaster*, in which repellent compounds were shown to reduce the attraction to food-related odors when repellent and attractive compounds are mixed [31]. The disturbed response to the *T. confusum* aggregation pheromone signal by *V. globosa* components could add a supplementary effect with respect to potential application. Inhibition of conspecific attraction could be synergistic to the direct repulsive effect of *V. globosa* oil and thus increase its efficiency.

Behavioral tests showed that anethole elicited a strong repellent effect, similarly to the complete essential oil at a dose of 0.1 mg. However, caryophyllene, a second major compound present in *V. globosa* at 7.72%, only showed a repellent effect at the highest tested dose of 1 mg, but the combination of the two major compounds of *V. globosa* (caryophyllene and anethole) are as strongly repellent as the whole essential oil. This suggests that the large quantity of anethole present in *V. globosa* (41.53%) might be at the origin of the repellent effect of the complete essential oil. This does, however, not exclude that other compounds, such as spathulenol or even minor non-identified compounds also contribute to the repellent activity of the essential oil and can work in synergy. Synergistic interactions of essential oil compounds have been described earlier in different moth species in the context of feeding deterrence [32], and in toxicity tests, where major constituents in a mixture can be more toxic than when tested individually [32,33,34].

EAG recordings revealed that the two major components, their blend, and the complete essential oil, elicited an antennal response. However, the antennae of *T. confusum* adults detect the essential oil and its major compounds at different doses. The lowest response thresholds were observed for the detection of the complete *V. globosa* essential oil in *T. confusum* antennae. Individual compounds elicited responses only at 1000-fold higher doses in the stimulation pipette, whereas the blend of the two individual compounds elicited a response at an intermediate dose. Another stored product pest insect, *Tenebrio molitor,* displayed strong EAG responses to various essential oils and some of their constituents, cis-3-hexenol, isoeugenol, α-pinene, turpentine oil, eucalyptus oil, and peppermint essential oil [35]. In *T. castaneum*, females and males exhibited similar EAG responses and responded strongly to undecane, 1-hexen-3-ol, octanal, 2-heptanone, hexanoic acid, and ethyl hexanoate [36].

In our study, caryophyllene is detected with the same sensitivity as anethole by the insect but induced a repellent effect in the behavioral test only at a higher dose. The detection of plant compounds or pheromones via olfactory receptors in phytophagous insects does not necessarily imply an important role for these cues in attraction or repulsive responses [37,38]. For example, the compounds limonene, citronellol, and nerol, elicited strong and moderate EAG responses in stable flies, but they elicited only low behavioral repellency [39].

In conclusion, our work shows that *V. globosa* essential oil as a whole or its major compound anethole could potentially be used in the development of alternative pest management strategies for the protection of stored products against *T. confusum.* Essential oils of other plants containing anethole such as star anise or fennel could be a good alternative, because of the low yield of *V. globosa* essential oil (see for example [40]). Further investigations are, however, needed to develop potential treatment strategies, for example by treating storage surfaces [41]. It would also be interesting to test the toxicity of *V. globosa* essential oil and anethole on *T. confusum* in order to collect more information on its potential use in alternative pest management. The low yield of essential oil from *V. globosa* leaves does most likely not allow large-scale production of the oil, thus the use of commercially available anethole alone or other plant essential oils containing anethole might be more promising.

## 4. Materials and Methods

### 4.1. Insects

*T. confusum* adults used in our experiments were reared in the laboratory of Agrocampus Ouest, Angers and came from a culture established at the University of Tizi-Ouzou in Algeria, which was transferred to Agrocampus Ouest in Angers in 2019. Insects were kept in glass containers filled with semolina. The containers were maintained in a dark chamber under a constant temperature of 30 ± 2 °C and relative humidity of 70 ± 5%.

Because earlier studies did not detect important differences in behavioral and electrophysiological responses between males and females [5,42], and because sexing is only possible at the pupal stage with a high error rate, we used non-sexed insects aged less than 3 months for all experiments. Insects for all experiments were taken from the mass rearing containers 1 h before their use in the olfactometer or electroantennogram (EAG) setup. Freshly hatched adults, which were colorless and less mobile than older individuals, were avoided in the tests.

### 4.2. Plants, Essential Oils and Individual Compounds

Leaves of *V. globosa* were collected in Caatinga vegetation in Alagoinha country in the state of Pernambuco (Brazil). The collection site is located at latitude 08°27′59″ south and at longitude 36°46′33″ west. Taxonomic identification was carried out by experts at the Instituto Agronômico de Pernambuco (IPA) and the voucher specimens were deposited at the herbarium under the reference number 93.137. Legal registration for accessing and studying Brazilian genetic heritage was done in the Sistema Nacional de Gestão do Patrimônio Genético e do Conhecimento Traditional Associado (SisGen-A80E074). The material was hydrodistilled using a Clevenger apparatus to obtain the essential oil. Twenty grams of dried (in an oven with air circulation at 60 °C for 48 h) and crushed leaves were placed in 250 mL of de-ionized water with some pumice stones and the distillation procedure took 3 h. We recovered the essential oil droplet in 1 mL of xylene.

Anethole (99% purity) and caryophyllene (>98.5% purity) were provided by Sigma-Aldrich (Saint-Quentin Fallavier, France).

All stimuli were diluted in mineral oil and 10 μL of each solution was applied on a filter paper inserted into a Pasteur pipette. Pure mineral oil was used as a control stimulus.

### 4.3. Gas Chromatography-Mass Spectrometry

The essential oil was analyzed on a gas chromatograph-mass spectrometer (GCMS-QP2010 SE, Shimadzu, France). The separation was performed on a ZB-5MS capillary column (30 m × 0.25 mm × 0.25 μm, Phenomenex, France) and helium was employed as the carrier gas with a flow rate of 1.1 mL/min. The oven temperature was set to 60 °C, held for 6 min, then a temperature gradient at 1 °C per min was applied up to 220 °C and held for 4 min. The injector, interface, and source ionization temperatures were set at 220 °C. The essential oil was diluted in 1 mL of xylene and 1 μL was injected in split mode with a split ratio of 150. The ionization (electronic impact) was performed at 70 eV.

Compounds were identified by comparing mass spectra with commercially available mass spectrometry libraries (NIST11, NIST11s, and FFNSC2) and by using standards. The relative amounts of the different compounds were calculated by determining the areas under the curve of the chromatographic peaks.

### 4.4. Olfactometer Tests

Four-way olfactometers for small insects [43] were used for all behavioral choice tests, as described earlier [9]. Briefly, a total airflow of 500 mL/min was applied to a central hole in the device, by a sucking membrane pump, resulting in a 125 mL/min flow in each branch. Five mL syringes containing Pasteur pipettes with the test stimulus applied to a piece of filter paper were connected to the inlets of the olfactometer and diagonal inlets received the same stimulus (test odor or solvent control). Insects were introduced individually into the olfactometer through the central hole, and the time spent in each of the four odorized areas was recorded during 15 min under low daylight conditions (150–300 lux). Olfactometers were cleaned with detergent after each experiment. The orientation of the olfactometer branches was changed after each experiment to avoid a potential bias of the experimental setup. At least 28 insects were tested for each treatment and experiments for the same treatment were performed on at least 2 different experimental days.

#### 4.4.1. Behavioral Experiments for Single Odors

Control experiments. To make sure that there was no bias in the experimental procedure, we performed control experiments applying the solvent mineral oil used to dilute our different odorants on all four branches.

Experiment 1. *V. globosa* essential oil was diluted in mineral oil and four final amounts on filter paper were used (5 mg, 1 mg, 0.1 mg, and 0.01 mg). Ten µL of mineral oil was used as control.

Experiment 2. Two major compounds of the essential oil of *V. globosa* were also tested individually. Anethole and caryophyllene represented respectively 41.52% and 7.72% of the oil. Final amounts on the filter paper of 0.01 mg, 0.1 mg, 0.4 mg, and 1 mg of anethole and 0.1 mg and 1 mg of caryophyllene were used. Dilutions were prepared in mineral oil and this solvent was used as a control stimulus.

Experiment 3. The attraction of *T. confusum* to conspecific aggregation pheromone was also evaluated. Five insects were placed in a silicone tube with Teflon mesh grids at both ends as stimuli [9]. The silicone tube was inserted in 5 mL syringe, similarly to the Pasteur pipette used for the other assays. Empty silicone tubes were used as control stimuli.

#### 4.4.2. Behavioral Experiments for Odor Combinations

Experiment 4. To test if anethole and caryophyllene have a synergistic repellent effect on *T. confusum*, we prepared a mixture of both compounds at the ratio of occurrence in the *V. globosa* essential oil (41.5/7.7). We prepared a solution containing 10 mg/µL of the mixture of compounds and diluted this solution to obtain 5 mg/µL, 1 mg/µL, and 0.1 mg/µL. We used 10 µL of each solution on the filter paper in behavioral tests. Mineral oil was used as a control stimulus.

Experiment 5. To examine if the essential oil is still repellent in the presence of aggregation pheromone emitted by *T. confusum*, we combined a silicon tube containing 5 beetles with a tube containing a filter paper with 5 mg of the *V. globosa* essential oil in the same syringe. The air passed first through the tube with the beetles and then through the essential oil-containing tube to avoid an influence of the essential oil on pheromone production. Mineral oil and an empty silicon tube were used as control stimuli.

### 4.5. Electroantennogram Recordings

EAG recordings were performed on whole insects, which had been starved overnight. The insect was fixed in a micropipette tip, with the thorax and head protruding. A small opening was cut in the thorax to insert the indifferent electrode and the antennal tip was cut and capped with a second glass capillary filled with Beadle Ephrussi Ringer. The electrodes were connected to an amplifier (axoclamp 2B, Molecular Devices, San Jose, CA, USA), and the signal was digitalized with an IDAC-4 device and recorded on EAG Pro software (Syntech, Kirchzarten, Germany). A constant airstream (0.3 m/s) was blown over the antenna through a glass tube. A stimulation pipette was inserted into a hole within the tube and a 300 ms air pulse was applied using a stimulation device (Stimulus controller CS 55, Syntech Kirchzarten, Germany). For each stimulus, five decadic doses on the filter paper were tested (from 0.1 µg to 1000 µg). The mixture of anethole and caryophyllene was prepared at the same ratio as for behavioral experiments. For each stimulus, mineral oil was tested before a series of increasing doses. At least one second elapsed between two consecutive stimuli. Response amplitudes for each stimulus were recorded and mineral oil responses were subtracted to obtain dose-response curves. Antennae from 17 insects were recorded for individual compounds and their mix. Antennae from 11 insects were recorded for the *V. globosa* oil.

### 4.6. Data Analysis

The statistical analyses were performed using XLSTAT software (Addinsoft, New York, NY, USA).

#### 4.6.1. Behavioral Experiments

The mean time spent in the two zones of the olfactometer with a test stimulus was compared to the mean time spent in the two zones with a control stimulus with a paired t-test. Data were tested for normality beforehand with Lilliefors and Jarque-Bera tests, revealing normal distribution.

#### 4.6.2. Electroantennogram Recordings

An ANOVA followed by Tukey’s post hoc test was performed to compare dose-response curves obtained in the EAG recordings to the different stimuli. Data were tested for normality beforehand with Lilliefors and Jarque-Bera tests, revealing normal distribution.

## Figures and Tables

**Table 1:** GC-MS analysis of *V. globosa* essential oil.

^1^ Compounds Similarity Index > 90% (NIST)	Retention Time (min)	Relative Percentage (%)
β-Pinene	8.73	0.17
Estragole	27.95	0.89
Anethole	38.28	41.53
Δ-Elemene	43.59	1.77
α-Copaene	47.9	0.59
β-Elemene	50.19	0.64
Caryophyllene	52.83	7.72
α-Humulene	56.90	2.6
γ-Gurjunene	59.96	0.56
β-Cubebene	60.36	3.04
β-Selinene	60.86	0.49
^2^ NI	62.04	1
Elixene	62.28	4.94
α-Guaiene	63.19	1.15
γ-Cadinene	64.56	0.57
^2^ NI	64.93	0.17
^2^ NI	65.08	1.77
Δ-Cadinene	65.92	1.2
Germacrene B	69.09	0.58
Spathulenol	71.86	7.06
^2^ NI	72.51	0.56
^2^ NI	74.50	2.08
Humulene epoxide II	75.14	0.32
^2^ NI	76.24	0.27
^2^ NI	76.89	1.58
^2^ NI	77.48	0.86
^2^ NI	78.18	6.69
^2^ NI	79.04	1.47
^2^ NI	79.98	0.54
^2^ NI	80.47	1
Cadin-4-en-10-ol	80.80	1.01
Shyobunol	84.36	3.53
^2^ NI	84.81	0.6
^2^ NI	88.17	0.62
(*E*,*E*)-Geranyllinalool	119.56	0.43

^1^ The compounds were identified by gas chromatograph-mass spectrometer (GCMS-QP2010 SE, Shimadzu). The separation was performed on a ZB-5 capillary column (30 m × 0.25 mm × 0.25 μm). ^2^ NI = Not Identified.

## Data Availability

Data sets of the present study have been deposited in zenodo https://zenodo.org/record/5027002#.YNSeQm46-ek, DOI 10.5281/zenodo.5027002.

## References

[1] Mohammed H.H. (2013). Repellency of Ethanolic Extract of Some Indigenous Plants against *Tribolium confusum* (Coleoptera: Tenebrionidae. J. Agric. Vet. Sci..

[2] Javadzadeh M., Sheikhi-Garjan A., Hosseini-Gharalari A. (2017). Susceptibility of Different Populations of *Tribolium confusum* (Coleoptera: Tenebrionidae) to Malathion (EC 57%) in Flour Mills of Iran. Acta Phytopathol. Entomol. Hung..

[3] Baker T.C. (1989). Sex Pheromone Communication in the Lepidoptera: New Research Progress. Experientia.

[4] Byers J.A. (1989). Chemical Ecology of Bark Beetles. Experientia.

[5] Verheggen F., Ryne C., Olsson P.O.C., Arnaud L., Lognay G., Högberg H.E., Persson D., Haubruge E., Löfstedt C. (2007). Electrophysiological and Behavioral Activity of Secondary Metabolites in the Confused Flour Beetle, *Tribolium confusum*. J. Chem. Ecol..

[6] Gadenne C., Barrozo R.B., Anton S. (2016). Plasticity in Insect Olfaction: To Smell or Not to Smell?. Annu. Rev. Entomol..

[7] Renou M., Anton S. (2020). Insect Olfactory Communication in a Complex and Changing World. Curr. Opin. Insect Sci..

[8] Zhang R., Wang B., Grossi G., Falabella P., Liu Y., Yan S., Lu J., Xi J., Wang G. (2017). Molecular Basis of Alarm Pheromone Detection in Aphids. Curr. Biol..

[9] Kheloul L., Kellouche A., Bréard D., Gay M., Gadenne C., Anton S. (2019). Trade-off between Attraction to Aggregation Pheromones and Repellent Effects of Spike Lavender Essential Oil and Its Main Constituent Linalool in the Flour Beetle *Tribolium confusum*. Entomol. Exp. Appl..

[10] Rodrigues F.A., Pimenta V.d.S.C., Braga K.M.d.S., de Araújo E.G. (2016). Obtenção de extratos de plantas do cerrado. Enciclopédia Biosf..

[11] De Oliveira J.V., de França S.M., Barbosa D.R.eS., Dutra K.d.A., de Araujo A.M.N., Navarro D.M.d.A.F., de Oliveira J.V., de França S.M., Barbosa D.R.eS., Dutra K.d.A. (2017). Fumigation and Repellency of Essential Oils against *Callosobruchus maculatus* (Coleoptera: Chrysomelidae: Bruchinae) in Cowpea. Pesqui. Agropecuária Bras..

[12] Bakkali F., Averbeck S., Averbeck D., Idaomar M. (2008). Biological Effects of Essential Oils—A Review. Food Chem. Toxicol..

[13] Barreca S., La Bella S., Maggio A., Licata M., Buscemi S., Leto C., Pace A., Tuttolomondo T. (2021). Flavouring Extra-Virgin Olive Oil with Aromatic and Medicinal Plants Essential Oils Stabilizes Oleic Acid Composition during Photo-Oxidative Stress. Agriculture.

[14] Gaire S., Scharf M.E., Gondhalekar A.D. (2020). Synergistic Toxicity Interactions between Plant Essential Oil Components Against the Common Bed Bug (*Cimex lectularius* L.). Insects.

[15] Sharma K., Guleria S., Razdan V.K., Babu V. (2020). Synergistic Antioxidant and Antimicrobial Activities of Essential Oils of Some Selected Medicinal Plants in Combination and with Synthetic Compounds. Ind. Crops Prod..

[16] Regnault-Roger C., Vincent C., Arnason J.T. (2012). Essential Oils in Insect Control: Low-Risk Products in a High-Stakes World. Annu. Rev. Entomol..

[17] Bougherra H.H., Bedini S., Flamini G., Cosci F., Belhamel K., Conti B. (2015). *Pistacia entiscus* Essential Oil Has Repellent Effect against Three Major Insect Pests of Pasta. Ind. Crops Prod..

[18] Chiluwal K., Kim J., Bae S.D., Park C.G. (2017). Essential Oils from Selected Wooden Species and Their Major Components as Repellents and Oviposition Deterrents of *Callosobruchus chinensis* (L.). J. Asia-Pac. Entomol..

[19] Oza M.J., Kulkarni Y.A. (2017). Traditional Uses, Phytochemistry and Pharmacology of the Medicinal Species of the Genus *Cordia* (Boraginaceae). J. Pharm. Pharmacol..

[20] Miguel M., Garcia-Bores A., Meraz S., Piedra E., Avila M., Serrano R., Orozco J., Jimenez-Estrada M., Chavarria J.C., Penalosa I. (2016). Antimicrobial Activity of Essential Oil of *Cordia globosa*. Afr. J. Pharm. Pharmacol..

[21] de Menezes J.E.S.A., Lemos T.L.G., Silveira E.R., Pessoa O.D.L., Santiago G.M.P., Nascimento R.F. (2006). Chemical Composition and Larvicidal Activity of the Essential Oil From Leaves of *Cordia globosa* (Jacq.) H.B.K. from Northeastern Brazil. J. Essent. Oil Res..

[22] de Carvalho P.M., Rodrigues R.F.O., Sawaya A.C.H.F., Marques M.O.M., Shimizu M.T. (2004). Chemical Composition and Antimicrobial Activity of the Essential Oil of *Cordia verbenacea* D.C. J. Ethnopharmacol..

[23] Hernandez T., Canales M., Teran B., Avila O., Duran A., Garcia A.M., Hernandez H., Angeles-Lopez O., Fernandez-Araiza M., Avila G. (2007). Antimicrobial Activity of the Essential Oil and Extracts of *Cordia curassavica* (Boraginaceae). J. Ethnopharmacol..

[24] Mhamdi B., Wannes W.A., Dhiffi W., Marzouk B. (2009). Volatiles From Leaves and Flowers of Borage (*Borago officinalis* L.). J. Essent. Oil Res..

[25] Zribi I., Bleton J., Moussa F., Abderrabba M. (2019). GC-MS Analysis of the Volatile Profile and the Essential Oil Compositions of Tunisian *Borago officinalis* L.: Regional Locality and Organ Dependency. Ind. Crops Prod..

[26] Fiori J., Hudaib M., Valgimigli L., Gabbanini S., Cavrini V. (2002). Determination of Trans-Anethole in *Salvia sclarea* Essential Oil by Liquid Chromatography and GC-MS. J. Sep. Sci..

[27] Senatore F., Oliviero F., Scandolera E., Taglialatela-Scafati O., Roscigno G., Zaccardelli M., De Falco E. (2013). Chemical Composition, Antimicrobial and Antioxidant Activities of Anethole-Rich Oil from Leaves of Selected Varieties of Fennel [*Foeniculum vulgare* Mill. Ssp. vulgare Var. azoricum (Mill.) Thell]. Fitoterapia.

[28] Tunç I., Erler F. (2000). Fumigant Activity of Anethole, a Major Component of Essential Oil of Anise *Pimpinella anisum* L.. Integr. Prot. Stored Prod. IOBC Bull..

[29] Alkan M., Ertürk S. (2020). Insecticidal Efficacy and Repellency of Trans-Anethole Against Four Stored-Product Insect Pests. J. Agric. Sci..

[30] Cao J.-Q., Guo S.-S., Wang Y., Pang X., Geng Z.-F., Du S.-S. (2018). Contact Toxicity and Repellency of the Essential Oils of *Evodia lenticellata* Huang and *Evodia rutaecarpa* (Juss.) Benth. Leaves against Three Stored Product Insects. J. Oleo Sci..

[31] Thoma M., Hansson B.S., Knaden M. (2014). Compound Valence Is Conserved in Binary Odor Mixtures in *Drosophila melanogaster*. J. Exp. Biol..

[32] Koul O., Singh R., Kaur B., Kanda D. (2013). Comparative Study on the Behavioral Response and Acute Toxicity of Some Essential Oil Compounds and Their Binary Mixtures to Larvae of *Helicoverpa armigera, Spodoptera litura* and *Chilo partellus*. Ind. Crops Prod..

[33] Jiang Z., Akhtar Y., Bradbury R., Zhang X., Isman M.B. (2009). Comparative Toxicity of Essential Oils of *Litsea pungens* and *Litsea pubeba* and Blends of Their Major Constituents against the Cabbage Looper, *Trichoplusia ni*. J. Agric. Food Chem..

[34] Chen Y., Luo J., Zhang N., Yu W., Jiang J., Dai G. (2021). Insecticidal Activities of *Salvia hispanica* L. Essential Oil and Combinations of Their Main Compounds against the Beet Armyworm *Spodoptera exigua*. Ind. Crops Prod..

[35] Wang Y., Li P., Chi D. (2016). Electrophysiological and Behavioral Responses of *Tenebrio molitor* L. to Fourteen Kinds of Plant Volatiles. J. Asia-Pac. Entomol..

[36] Balakrishnan K., Holighaus G., Weißbecker B., Schütz S. (2017). Electroantennographic Responses of Red Flour Beetle *Tribolium castaneum* Herbst (Coleoptera: Tenebrionidae) to Volatile Organic Compounds. J. Appl. Entomol..

[37] Badji C.A., Eiras A.E., Cabrera A., Jaffe K. (2003). Avaliação Do Feromônio Sexual de *Neoleucinodes elegantalis* Guenée (Lepidoptera: Crambidae). Neotrop. Entomol..

[38] Bruce T.J.A., Wadhams L.J., Woodcock C.M. (2005). Insect Host Location: A Volatile Situation. Trends Plant Sci..

[39] Hieu T.T., Jung J., Kim S.-I., Ahn Y.-J., Kwon H.W. (2014). Behavioural and Electroantennogram Responses of the Stable Fly (*Stomoxys calcitrans* L.) to Plant Essential Oils and Their Mixtures with Attractants. Pest Manag. Sci..

[40] Destro B.G.I., Jorge R.M.M., Mathias A.L. (2019). Optimization of High-Concentration Trans-Anethole Production through Hydrodistillation of Star Anise. Braz. J. Chem. Eng..

[41] Arthur F.H. (1996). Grain Protectants: Current Status and Prospects for the Future. J. Stored Prod. Res..

[42] Suzuki T., Sugawara R. (1979). Isolation of an Aggregation Pheromone from the Flour Beetles, *Tribolium castaneum* and *T. confusum* (Coleoptera: Tenebrionidae). Appl. Entomol. Zool..

[43] Pettersson J. (1970). An Aphid Sex Attractant. Insect Syst. Evol..

